# Internal medicine residency training for unhealthy alcohol and other drug use: recommendations for curriculum design

**DOI:** 10.1186/1472-6920-10-22

**Published:** 2010-03-15

**Authors:** Angela H Jackson, Daniel P Alford, Catherine E Dubé, Richard Saitz

**Affiliations:** 1 Residency Training Program in Internal Medicine, Boston University School of Medicine, Boston, MA, USA; 2 Clinical Addiction Research and Education (CARE) Unit, Section of General Internal Medicine, Boston University School of Medicine, Boston, MA, USA; 3 Department of Medicine, Boston Medical Center, and Boston University School of Medicine, Boston, MA, USA; 4 Youth Alcohol Prevention Center, Boston University School of Public Health, Boston, MA, USA; 5 Department of Epidemiology, Boston University School of Public Health, Boston, MA, USA

## Abstract

**Background:**

Unhealthy substance use is the spectrum from use that risks harm, to use associated with problems, to the diagnosable conditions of substance abuse and dependence, often referred to as substance abuse disorders. Despite the prevalence and impact of unhealthy substance use, medical education in this area remains lacking, not providing physicians with the necessary expertise to effectively address one of the most common and costly health conditions. Medical educators have begun to address the need for physician training in unhealthy substance use, and formal curricula have been developed and evaluated, though broad integration into busy residency curricula remains a challenge.

**Discussion:**

We review the development of unhealthy substance use related competencies, and describe a curriculum in unhealthy substance use that integrates these competencies into internal medicine resident physician training. We outline strategies to facilitate adoption of such curricula by the residency programs. This paper provides an outline for the actual implementation of the curriculum within the structure of a training program, with examples using common teaching venues. We describe and link the content to the core competencies mandated by the Accreditation Council for Graduate Medical Education, the formal accrediting body for residency training programs in the United States. Specific topics are recommended, with suggestions on how to integrate such teaching into existing internal medicine residency training program curricula.

**Summary:**

Given the burden of disease and effective interventions available that can be delivered by internal medicine physicians, teaching about unhealthy substance use must be incorporated into internal medicine residency training, and can be done within existing teaching venues.

## Background

Unhealthy substance use (SU) is the spectrum from use that risks harm, to use associated with consequences or problems, to the diagnosable conditions substance abuse and dependence often referred to as substance use disorders [1]. Unhealthy SU is a major public health problem in the United States. Many physician interventions (e.g., brief counseling, pharmacotherapy) have proven efficacy. Internal medicine physicians are among the most commonly visited physicians in the US [2]. Yet internal medicine physician training in substance use-related preventive services, diagnosis, treatment, and chronic disease management has been inadequate. This inadequacy leaves patients and the health system without sufficient expertise to address one of the most common and costly health conditions.

Among people 12 and older, there were 20.4 million current users of illicit drugs, 125 million users of alcohol and 72.9 million users of tobacco products, according to the 2006 National Survey on Drug Use andHealth [3]. Of those, 22.6 million alcohol and illicit drug users (9.2% of the population 12 and older) met criteria for substance abuse or dependence. Drug abuse was responsible in 2002 for approximately 26,000 deaths and cost society $180.8 billion [4]. Alcohol use cost society similarly and was responsible for 85,000 deaths [5,6]. Comparatively, coronary heart disease, the leading cause of death in the United States for the past 80 years and a major cause of disability, cost an estimated $151.6 billion in 2007 [7,8].

Education about unhealthy SU is inadequate in medical training [9]. This deficiency persists despite the contribution of SU to disability and premature death, [10] and its prevalence and societal costs [6,11,12]. Screening and management of SU merits a position in medical curricula that reflects its importance and characteristics as a mainstream medical condition [13-17]. Although screening and brief intervention for unhealthy alcohol use is among the most effective and cost-effective preventive services delivered by physicians, its actual delivery is the lowest among comparably ranked services (most often not delivered to those eligible) [18-20].

Many physicians fail to address SU conditions due to discomfort with SU-related patient discussions, [21] deficient knowledge and clinical skills, [22,23] and negative attitudes, [24,25] all resulting in barriers to providing optimal medical care for their patients and reducing the consequences that affect their families and society [26]. The diagnosis of substance abuse or dependence is often missed by physicians and even when the diagnosis is made, many physicians do not know how to respond appropriately using brief intervention or developing an organized plan for referral or treatment and follow-up. While there are many reasons physicians are not performing screening and brief intervention, such as stigma or lack of skill, there may be few local referral resources for patients with SU, once identified. At minimum, the basic clinical skills of screening, assessment, diagnosis, negotiating treatment and ongoing monitoring in SU must be addressed in physician training. These are skills that physicians already routinely employ in the prevention and management of other chronic conditions [27]. SU conditions can be serious and chronic, and risk factors and earlier stage unhealthy use can be recognized, highlighting the need for physicians to embrace their role in preventing, identifying and managing patients with unhealthy SU [28].

## Discussion

### Physician education

Medical educators have started addressing the need for physician training in unhealthy SU screening, assessment, and management [29-34]. Formal curricula on these subjects have been developed [35,36] and evaluated [37,38] and recommendations for the medical care of addicted patients have been published [13,39,40] Nonetheless, dissemination of up-to-date addiction research and clinical recommendations into physician practice and residency curricula remains a significant challenge [41,42].

Unhealthy substance use education aimed at improving residents' attitudes and clinical practice behaviors has been shown to be effective [43,44]. When residents feel responsible for caring for patients with SU conditions (i.e., "role responsibility"), they develop greater confidence in their ability to screen and refer patients [45]. Wider implementation of known effective clinical practices for addressing SU conditions requires creative strategies to develop a workforce that sees the management of SU conditions as part of its overall mission, is knowledgeable about state-of-the-art approaches to patient management, and is motivated to implement such practices in a range of clinical settings [9,38,46,47]. As noted in the Institute of Medicine Report *Improving the Quality of Health Care for Mental and Substance-Use Conditions*, [26] medical educators have not adequately addressed past recommendations to update training of medical professionals, leaving trainees ill equipped in their ability to care for patients with SU conditions.

The need to implement SU curricula is also supported by the existence of several national initiatives regarding SU care in medical settings--the Joint Commission, which is considering SU-related performance measures for hospitals, a performance measure for alcohol screening included by the Center for Medicare and Medicaid Services in 2009, and national Screening Brief Intervention Referral and Treatment programs supported by federal grants to a number of states in the US [48-50].

### Strategies for educational change

There are several strategies that may be employed to foster the adoption of core addiction medicine competencies into mainstream of graduate medical education curricula, each with strengths and limitations. Examples include:

1) **Modifying residency training to support the development of core skills and behaviors **by the program graduates, though residency programs may be reluctant to add new training initiatives to their busy schedules;

2) **Disseminating models for understanding SU conditions that are already familiar to physicians**, for example, highlighting that SU conditions are often chronic diseases with periods of remission and relapse for many patients; [51]

3) **Addressing attitudes towards unhealthy SU and patients with these conditions**, recognizing that attitudinal issues play a large role in physicians' willingness to address SU conditions in their patients. For example, clinical guidelines and protocols may be more readily accepted if championed by opinion leaders and role models who are trusted sources of clinical information (often requiring them to be from the same specialty and profession), effective presenters of new information about changes in clinical practice and viewed as mentors by colleagues and younger trainees;

4) **Recommendations and requirements of accreditation bodies **to serve as catalysts and ultimately for enforcement of change within training programs.

### Accreditation and certification to improve resident physician unhealthy substance use education

Academic institutions provide learners with opportunities to develop knowledge and skills that are pre-requisites for safe, effective, and competent practice. Accrediting organizations assess educational *programs *to determine whether their content is designed to produce fully competent graduates. Accreditation is granted to those programs meeting their standards. The Accreditation Council for Graduate Medical Education (ACGME) is a private, non-profit council that evaluates and accredits medical residency programs in the United States. The ACGME was established in 1981 based on a consensus in the academic medical community for an independent accrediting organization. Its forerunner was the Liaison Committee for Graduate Medical Education (LCGME) and had been established in 1972.

The mission of the ACGME is to improve health care by assessing and advancing the quality of resident physicians' education through accreditation. For each medical specialty, the ACGME has a Residency Review Committee (RRC) comprised of 6 to 15 volunteer physicians. Members of the residency review committees are appointed by the American Medical Association (AMA) Council on Medical Education and the appropriate medical specialty boards and organizations.

In the evaluation of graduate medical education, the ACGME has shifted from a *descriptive model *focused on structure and measurement of a program's "potential" to train competent physicians, to a model that measures actual training *outcomes*. In 1997, the ACGME initiated the Outcome Project and began to develop core competencies. The goal of the Outcomes Project is to enhance residency education through resident outcome assessment [52]. This project is a long-term initiative which emphasizes the attainment of a core set of competencies by the residents, as an indicator of a residency program's educational effectiveness and quality rather than simple compliance with regulations. In 1999, the AGGME endorsed six general competencies around which all residency curricula should be organized: 1) Medical Knowledge; 2) Patient Care; 3) Interpersonal and Communication Skills; 4) Professionalism; 5) Practice-based learning and improvement; 6) Systems-based practice. The ACGME has progressively moved to the present mandate for training programs to demonstrate data-driven changes and improvements in curricula based on resident performance data in each of the competencies, promoting continuous improvement in resident education and ultimately, in the healthcare workforce. Any efforts to improve resident physician unhealthy substance use education via accreditation will likely be most successful if they take into account and relate clearly to the ACGME core competencies.

Health professional organizations frequently rely on independent certifying bodies that grant certification recognizing that individuals have successfully demonstrated knowledge or competency in a particular specialty. The American Board of Internal Medicine (ABIM) is a non-profit, independent evaluation organization that, through the administration of a certifying examination, has for more than 70 years maintained the highest standard in internal medicine. ABIM certification has meant that internists have demonstrated - to their peers and to the public - that they have the clinical judgment, skills and attitudes essential for the delivery of excellent patient care. However, only 2% of the American Board of Internal Medicine certifying exam typically addresses substance use, which translates into 2-5 questions in the entire exam (compared to 14% for cardiovascular disease, 6% for nephrology, 2% for ophthalmology, and 10% for geriatrics). Regulatory bodies may provide some leverage in instituting more global implementation of resident training in substance abuse conditions by increasing the emphasis of substance use and related conditions on their examinations, such as on the ABIM certifying examination.

### Unhealthy substance use-related competencies

The Health Resources and Services Administration (HRSA) and the Substance Abuse and Mental Health Services Administration (SAMHSA) Center for Substance Abuse Treatment (CSAT) supported an effort by the Association for Medical Education and Research in Substance Abuse (AMERSA) to implement an interdisciplinary project to improve health professional education in substance abuse [53]. The project was known as Project MAINSTREAM (the Multi-Agency INitiative on Substance abuse Training and Education for America).

A major aim of this project was to produce a national strategic plan to improve care for substance use problems, including state-of-the-art reviews and recommendations for health professional development by leading authorities. To develop the strategic plan, nationally recognized experts were invited to join a Strategic Planning Advisory Committee (SPAC) representing dentists, dietitians, nurse midwives, nurses, nurse practitioners, occupational therapists, pharmacists, physical therapists, physicians, physician assistants, psychologists, public health professionals, rehabilitation counselors, social workers, speech pathologists, and audiologists.

Using a modified consensus-development approach, they defined a set of core competencies for all health professionals, irrespective of discipline. In addition, members of the SPAC, in conjunction with other national leaders in substance abuse, developed discipline-specific papers that summarize the state of the art regarding education of health professionals about SU conditions and provide recommendations and action steps for achieving desired goals within each discipline. All of the papers were subjected to peer review and were modified before being accepted for inclusion in the Strategic Plan. Following further review of the papers, an exhaustive stratification process was used to derive key recommendations that cut across the professional disciplines represented by the authors. The recommendations represent the collective input from SPAC members and outside experts from all of the disciplines and hundreds of other individuals who assisted in the review of materials in the Strategic Plan.

Since publication of the Strategic Plan in 2002, recommended physician competencies were adopted by the White House Office of National Drug Control Policy and by medical education leaders (including representatives from the ACGME, AMA, and the Society of General Internal Medicine) in a series of Leadership Conferences on Medical Education in Substance Abuse that took place in 2004, 2006, and twice in 2008, [28]

#### Unhealthy Substance Use Curriculum for Internal Medicine Residency Programs

##### Introduction to Unhealthy Substance Use Curriculum

Many previous publications have outlined curricula for physicians at various stages of training and from various specialties, for medical schools and residencies. In this paper, we outline a curriculum in unhealthy substance use education for internal medicine resident physicians specifically, based on the core competencies developed as outlined above. We provide an outline to assist in the actual implementation of such a curriculum within an internal medicine residency training program.

We have organized the curriculum into modules, with didactic as well as experiential components, utilizing a variety of educational venues, some new and some typically found within the existing framework of an internal medicine residency curriculum. Residency training programs may opt to deliver this curriculum via a dedicated rotation. While a dedicated rotation may be more efficient, it may also be less likely to be implemented as required components of residencies already replete with such rotations (e.g., intensive care unit). More importantly, we believe that addiction medicine is best taught to medical residents when the training is *integrated *into general medical care, modeling comprehensive care delivery. In this format, components of the unhealthy substance use curriculum are inserted into existing internal medicine teaching venues, both didactic and clinical, and the competencies contribute to the core general competencies addressed by residencies and monitored by the ACGME.

This model relies heavily on faculty who are well trained in addiction medicine and can serve as effective teachers. Such a model may also provide only limited exposure to patients in recovery after having received specialty treatment, who are less often recognized in internal medicine clinical settings. Finally, we link the proposed curricular modules to the ACGME core competencies. These modules may be modified and adapted to meet specific program needs and available resources.

##### Goal of the unhealthy substance use curriculum

The goal of an unhealthy substance use (SU) curriculum for internal medicine residents is two-fold. The first goal is to highlight the importance of addiction medicine in patient care. The second is to provide internal medicine residents, regardless of ultimate career choice, with the core knowledge and skills necessary for all internists who provide clinical care. Of note, internists include those in general internal medicine (many of whom deliver primary medical care) as well as subspecialists (e.g., cardiologists, gastroenterologists, endocrinologists, nephrologists). Unhealthy SU condition knowledge and skills address appropriate prevention, early detection, diagnosis, treatment and referral for patients with substance use conditions. We outline herein a curriculum in unhealthy SU for internal medicine residents, based on the recommendations of AMERSA's Project MAINSTREAM regarding physician competencies. These **core competencies in unhealthy substance use for internal medicine residents **are as follows:

1) Residents will perform age, gender and culturally appropriate unhealthy substance use screening

2) Residents will effectively assess patients with unhealthy substance use

3) Residents will provide brief interventions to patients with unhealthy substance use

4) Residents will demonstrate effective counseling methods to help prevent unhealthy substance use

5) Residents will refer patients with substance use disorders to treatment settings that provide pharmacotherapy for relapse prevention

6) Residents will recognize, treat or refer co-morbid medical and psychiatric conditions in patients with substance use conditions

7) Residents will refer patients with substance use disorders to appropriate treatment and supportive services

8) Residents will be aware of the ethical and legal issues around physician impairment from substance use and of resources for referring potential impaired colleagues, including employee assistance programs, hospital based committees, and state physician health programs and licensure boards

9) Residents will identify the legal and ethical issues involved in the care of patients with unhealthy substance use

10) Residents will provide pharmacologic withdrawal to patients with substance dependence

11) Residents will provide or refer for treatment for relapse prevention in patients with substance use disorders, both pharmacotherapy and psychosocial counseling

##### Unhealthy substance use curriculum

The curriculum is presented in eight modules, outlined in Table 1. The modules address the substance use competencies outlined by Project MAINSTREAM and are linked to the ACGME competencies that they can meet. Table 1 suggests where to integrate the modules into an internal medicine residency program's existing clinical venues and an anticipated minimum time for each. The didactic sessions for each of the eight modules listed in Table 1 may be appropriate for more than one didactic session depending on the depth of teaching and the availability of group learning conference time. The curriculum is presented in separate modules, as a suggestion for implementation. However, these may be modified based on the needs of the individual training programs. For example, a residency program might opt to consolidate the screening and brief intervention modules into one didactic session, followed by a skill practice workshop.

**Table 1:** Unhealthy substance use curricular modules, corresponding ACGME competencies and suggested internal medicine residency clinical venues

Modules	ACGME Competencies	Suggested Clinical Venue	Time
1) Addiction and the brain: principles of addiction	Medical knowledge; Patient care	All patient care activities, especially continuity clinic experiences; inpatient medicine; emergency department	45-60 minute lecture

2) Complications and comorbidities of unhealthy substance use	Medical knowledge; Patient care; Systems-based practice	Medical consultation rotations; inpatient medicine; emergency department; intensive care unit rotations	45-60 minute case-based lecture

3) Screening and assessment of unhealthy substance use	Practice-based learning;	Continuity clinic; inpatient medicine; subspecialty electives; emergency department	45-60 minute interactive lecture and 45-60 minute skills practice session

4) Effective methods of counseling patients including brief intervention	Patient care Medical knowledge; Interpersonal and communication skills; Systems-based practice	Continuity clinic; inpatient medicine; emergency department	45-60 minute interactive lecture and 45-90 minute skills practice (1-2 sessions)

5) Substance abuse treatment including pharmacotherapy	Systems-based practice; Medical knowledge; Patient care	Intensive care units, medical wards, continuity clinics, emergency department	60-90 minute lecture

6) Substance-specific inpatient and outpatient management	Medical knowledge; Patient care; Practice-based learning; Systems-based practice; Interpersonal and communication skills	Continuity clinic; inpatient medicine; emergency department	60 minute case-based lecture

7) Prescription drug abuse	Professionalism; Interpersonal and communication skills; System-based practice; Practice-based learning; Patient care; Medical knowledge	Continuity clinic; emergency department	60 minute case-based lecture

8) Legal and ethical considerations for patients and physicians	Professionalism; Systems-based practice; Practice-based learning; Medical knowledge	All patient care activities; continuity clinic; inpatient medicine; emergency department	60 minute lecture

#### 1) Addiction and the brain: principles of addiction medicine

##### Module relevance

This introductory module sets the stage for covering substance use conditions as important and relevant, often chronic conditions, for generalists. It will teach learners the pathophysiology of addiction.

##### Module content

The session should introduce addiction as a chronic relapsing brain disease by reviewing the neurobiology of addiction. The neurobiology of addiction should include where in the brain substances of abuse act, how they cause intoxicating effects and how they alter the brain when used chronically. In this way, addiction medicine is framed similarly to how other chronic diseases (e.g., chronic obstructive lung disease, congestive heart failure, and diabetes) are taught. The session should also include national and local epidemiology of substances of abuse and describe the full spectrum of unhealthy substance use from at-risk use (that risks consequences) to substance dependence. A general overview about how the severity of the substance use problem influences treatment choice and efficacy and relapse risk should be covered. The session should be clinically relevant to the learner, for example, epidemiology and treatment should be presented in a way that is relevant to the specific residency program's needs and to the specific residents' rotation. For example, if the module is being taught to an inpatient team in an urban setting where heroin dependence is prevalent, then the prevalence, neurobiology and management of heroin dependence in hospitalized patients should be included. Discussion about the genetic vulnerabilities, risk and protective factors of addiction should also be covered.

##### Module special considerations

Depending on the population served by the residency program's clinical sites, the curriculum should address the specific needs for special populations (adolescent, geriatric, racial/ethnic/cultural groups) and for specific substances of abuse.

#### 2) Complications and comorbidities of substance abuse

##### Module relevance

This module serves to highlight the impact SU has on other common medical and psychiatric diseases. The module is also important because of the high prevalence of medical and psychiatric comorbidities in patients with substance use conditions. As such it has particular relevance for internal medicine physicians focused on medical conditions, and common psychiatric conditions such as depression and anxiety. Addressing substance use allows more effective management of these conditions.

##### Module content

It will be important for learners to understand how substance use causes or worsens other chronic diseases (e.g., cirrhosis, cardiomyopathy, depression) and has important interactions with treatments for other chronic diseases (e.g., anticoagulation therapy, sedatives for anxiety, opioids for chronic pain). This module highlights how common medical conditions (e.g., hypertension, insomnia) can be adversely affected by substance use. In addition, injection drug use and risky sexual behavior during substance use has been associated with conditions such as endocarditis, hepatitis B and C and HIV/AIDS. Topics such as cocaine associated chest pain or injection drug using patients with fever are useful contexts to present this topic.

#### 3) Screening and assessment for unhealthy substance use

##### Module relevance

This module has relevance for residents as screening and early intervention for unhealthy substance use are recommended practices for all adults[54]. Many internal medicine patients are unrecognized and once identified the problem can be addressed to prevent and manage substance use conditions. It also serves to teach specific skills on how to detect unhealthy (covering the spectrum from "at risk use" to "dependence") alcohol and drug use, using appropriate screening tools based on their validity, applicability and purpose.

##### Module content

This module should cover how unhealthy substance use meets the criteria for widespread screening based on high quality evidence (high prevalence, significant consequences, valid screening tests, effective and safe treatments, early identification and treatment are preferable). The evidence behind effective formal screening methods (rationale, utility, operating characteristics) should be covered. Learners should practice specific techniques (single item screening tests, quantity and frequency, CAGE, AUDIT, DAST) [1,55] demonstrating age, gender and culturally appropriate unhealthy substance use screening skills. Learners should appreciate the limitations of biological markers (e.g., urine drug testing, blood mean corpuscular volume, gamma-glutamyl-transferase, carbohydrate-deficient transferrin). This module should address steps to be taken to assess patient's severity of substance use and readiness to change their use in patients who screen positive. Using the stages of change model, learners should be able to assess a patient's readiness to change. Assessment should include identifying substance use disorders (e.g., whether the patient has dependence, or abuse, any consequences, or no consequences but excessive use). Teaching of assessment should also cover patient factors that increase the risk of any use, such as pregnancy or trying to conceive; medications contraindicated with substance use (e.g., warfarin); medical conditions that contraindicate alcohol or drug use (e.g., hepatitis); blackouts; failed attempts to cut down; family history of substance conditions; injuries related to substance use; medical conditions that may be caused by substance use (e.g., hypertension, trauma, anxiety, sleep disorders); and behavioral problems that can result from or be worsened by substance use (e.g., problems with work, school, or family).

#### 4) Effective methods of counseling patients including brief interventions

##### Module relevance

This module covers the effectiveness and skills development of counseling to help prevent the development of or progression of unhealthy substance use using formal psychological counseling and brief interventions. Brief counseling is one of the key skills in the recommended practice of screening and intervention. Motivational Interviewing and brief counseling are particularly important skills for managing patients with unhealthy substance use because many such patients do not recognize their condition, and when they do, they may not be ready to change. Brief counseling can facilitate change in this context. These skills also have relevance to internal medicine practice beyond addressing substance use, as they are useful for medication adherence, and behavior change counseling in general.

##### Module content

Residents should learn stages of change (precontemplation, contemplation, determination, action, relapse and maintenance) and appropriate counseling strategies including patient advice and education about harms and risks. Residents should learn the skills of patient centered motivational interviewing and how they differ from confrontational approaches. They should be able to apply the principles of motivational interviewing including developing discrepancy, avoiding argumentation, rolling with resistance, expressing empathy and supporting self efficacy. They should be able to ask open ended questions, listen reflectively, affirm, summarize and elicit and recognize change talk (i.e., disadvantages of the status quo, advantages of change, optimism for change or intention to change; desire, ability and reasons for change statements, and commitment language). Residents should also be skilled in helping to strengthen a patient's commitment to change by negotiating a plan. Residents should learn the skills of brief intervention (i.e., counseling) including the components of patient feedback, emphasizing personal responsibility for change, giving clear advice, giving a menu of treatment options, having an empathic counseling style and enhancing a patients self-efficacy.

##### Module special considerations

This module is best accomplished by employing skills practice where residents have a chance to role play brief intervention and motivational interviewing skills and receive feedback on their clinical skills.

#### 5) Substance Abuse Treatment including Pharmacotherapy

##### Module relevance

This module includes treatments that internal medicine physicians should have expertise in (e.g., pharmacotherapy for alcohol dependence) as well as treatments to which internists will generally refer patients (e.g., residential addiction specialty treatments).

##### Module content

The module covers the effectiveness and content of substance abuse treatments including detoxification, (i.e., medically supervised withdrawal) residential treatment, 12 step and mutual help programs, outpatient treatment and pharmacotherapy (e.g., methadone, buprenorphine, naltrexone, acamprosate). Residents should learn how to work collaboratively with substance abuse specialty treatment programs and clinicians and specialists including counselors, psychologists and social workers. Emphasis should be on talking to patients about specialty treatment, making appropriate referrals and prescribing medications to treat dependence. Residents should know the efficacy and limitations of different treatment modalities. They should be familiar with web-based substance abuse treatment locator resources (e.g., Substance Abuse and Mental Health Services Administration (SAMHSA) homepage http://www.samhsa.gov).

##### Module special considerations

Residents should have an opportunity to visit treatment programs (e.g., methadone maintenance program, alcoholics anonymous meeting) and interview patients who have undergone specialty treatment and/or attended mutual help groups, and those who are in recovery regardless of treatment history (many such patients can be found in the residents usual internal medicine training sites).

#### 6) Substance Specific Inpatient and Outpatient Management

##### Module relevance

This module serves to help residents identify specific substance intoxication and withdrawal syndromes, which are often seen in emergency, outpatient and inpatient medicine settings, and make evidence-based decisions on management strategies for specific substance (e.g., alcohol, opioid) intoxication, withdrawal and dependence.

##### Module content

This module should cover substance specific epidemiology, biochemistry, clinical pharmacology (e.g., pharmacokinetics, drug testing, drug-drug interactions), neurobiology, and behavioral effects (e.g., intoxication, tolerance, physical dependence). The major substance categories should be covered including central nervous system depressants, psychomotor stimulants, nicotine, opioids, cannabis and alcohol. Residents should be able to identify the signs and symptoms of intoxication, overdose and withdrawal of all the major categories of substances. Residents should learn how to manage acute intoxication and withdrawal syndromes of all the major categories of substances.

#### 7) Prescription Drug Abuse

##### Module relevance

The challenges of appropriate chronic pain treatment and recognition and prevention of prescription drug abuse are well known to internal medicine physicians. This module serves to give residents the knowledge and skills to prevent, and identify and manage a condition that is increasing in prevalence, prescription drug abuse (PDA).

##### Module content

This module should include an overview of PDA including epidemiology, and important definitions (e.g., prescription drug misuse, tolerance, physical dependence, aberrant medication taking behavior, pseudoaddiction). Residents should know which medications are more likely to be abused and factors that lead to physicians overprescribing controlled substances. Residents should learn a framework for safe prescribing including understanding when controlled substances are indicated, and what their efficacy is, screening patients for PDA risk, setting realistic therapeutic goals and monitoring strategies including urine drug testing, pill counts, use of patient agreements and informed consent and use of prescription monitoring programs. Residents should be able to identify prescription drug abuse and have the skills to communicate with patients about either the lack of benefit from the controlled substance or apparent harm (e.g,, the possible diagnosis of addiction) to controlled substances. While not specifically part of a substance use curriculum, this module should be complemented by education on the treatment of acute and chronic pain.

#### 8) Legal and Ethical Considerations for Patients and Physicians

##### Module relevance

Documentation of substance use and care for substance use occurs in internal medicine patient encounters from screening for unhealthy SU to referral and treatment for dependence. Care for patients with unhealthy SU often involves challenges related to family and employment which can raise ethical issues. As physicians competent to recognize unhealthy substance use, internists are in a position to recognize it in their colleagues who may need help. Residents should be aware of the ethical and legal issues around caring for patients with substance use conditions as well as issues around physician impairment.

##### Module content

This module should cover the patient confidentiality laws pertaining to managing patients with substance use conditions. Residents should also become aware of insurance coverage issues pertaining to substance use condition treatment and recognize that this coverage varies widely. Resources for information and updates on these topics should also be presented. Residents should be aware of the ethical and legal issues around physician impairment from substance use and of resources for referring potentially impaired colleagues including employee assistance programs, hospital-based committees, state physician health programs, and licensing boards.

#### Educational Venues

**There are two components to the recommended curriculum**-Didactic sessions and Clinical experiences.

##### Didactic sessions

The core topics on substance use conditions outlined in each module may be presented using the teaching conferences already in place in internal medicine residency programs (e.g., departmental grand rounds, morbidity and mortality conference, noon conferences, etc.). In fact, doing so has the distinct advantage of treating unhealthy substance use in the same way that other medical conditions are treated in the residency curriculum. The didactic sessions will address concepts that will be reinforced during clinical rotations, or will provide a venue to address issues of addiction medicine that the residents may not be directly exposed to in their clinical rotations. Examples of common internal medicine residency didactic venues that can be used for unhealthy substance use teaching, and how they might be used, are:

• *Lectures and Morbidity and Mortality Rounds/Conferences*: Overview of medical conditions with associated with substance abuse; overview of screening for substance abuse; detoxification procedures for alcohol and other drugs; medication management of addictions including relapse prevention; overview of appropriate prescribing practices for opioids and effective pain management strategies.

• *Grand Rounds*: As what is often the main academic conference within an entire department, this is an excellent venue for high profile, scientific presentations on unhealthy substance use.

• ***Case discussions***, *such as Morning Report (inpatient and ambulatory), attending rounds or continuity clinic conferences*: Topics in unhealthy substance use may be presented *de novo *or previously presented topics can be reinforced during these case discussions. This is accomplished by discussing not only patients' medical conditions, but also by highlighting the underlying substance use conditions that may be associated with (having caused or worsened) them. Examples include: hepatitis C (injection drug use), cardiomyopathy (alcohol), and rhabdomyolisis (cocaine). During case presentations, any aspect may be highlighted along the spectrum of unhealthy SU, integrating substance use management principles seamlessly into the clinical discussions.

• *Journal Clubs*: Presentations would focus on critical appraisal skills while also addressing and reinforcing concepts in addiction medicine, through review of peer-reviewed articles on unhealthy substance use in the medical literature. Examples include: studies of the effectiveness of brief interventions or use of buprenorphine.

Additional teaching opportunities for addressing unhealthy substance use beyond traditional internal medicine residency conferences include:

• *Quality Improvement (QI) Projects*: With the implementation of an unhealthy substance use curriculum, residents may have the opportunity to meet the ACGME requirement of a QI project, within their own continuity clinic practice, or inpatient experience. Projects related to an assessment of institutional or administrative systems affecting implementation of screening, treatment protocols for substance withdrawal, or availability of treatment referrals for their patients are examples.

• ***Workshops***: Programs that include workshops or seminars (e.g., multi-hour small group sessions) for skills development can incorporate skills practice sessions in motivational interviewing and brief counseling interventions, as well as in the use of screening tools and approaches to assessment.

• ***Field Trips***: Visits outside the medical residency training program clinic and hospital setting to 12-step meetings, [56] methadone and buprenorphine clinics, needle exchange programs, other substance abuse treatment programs broaden the residents' view of the spectrum of substance use conditions and their treatment. These activities can be incorporated into conference series, ambulatory block experiences and seminars, inpatient rotations, and electives.

• ***Meetings with patients in recovery***: Residents can hear first-hand accounts of effective and ineffective ways that physicians approached their substance use condition from the patients themselves, creating powerful learning experiences. They can also be exposed to patients who are no longer severely affected. This venue may be incorporated into resident clinic conferences, including small or large group teaching sessions.

• *Video-taped and other observed patient encounters*: Direct observation of a patient encounter is the "gold standard" to assess residents' attitudes, knowledge and skills in the area of patient-doctor communication, and can teach and assess skills in screening, motivational interviewing and brief intervention. Guided by a trained preceptor, video-taped review is a powerful learning experience.

##### Clinical experiences

Core concepts, presented in didactic sessions, can then be reinforced, and skills practiced, during residents' routine clinical rotations (inpatient medicine rotations, continuity clinics, emergency department, and intensive care unit rotations), complementing rather than replacing current curricular components, and delivered over the three years of training. Examples of experiential clinical learning experiences and opportunities include:

• *Inpatient hospital service rotations and intensive care rotations*: In these settings, residents can gain hands-on experience in the management of withdrawal syndromes (particularly opioids, alcohol and other sedatives), learn to assess the severity of addiction and readiness to change, and recognize and manage the complications and co-morbidities associated with substance use conditions, highlighting the importance of addressing the underlying substance use conditions. This is also a venue where resident physicians can have experiences and learning at the interface between pain and addiction.

• *Continuity clinics*: The resident continuity clinic is a site particularly well suited for the development of screening skills (and implementation--Practice-based learning), providing faculty with the opportunity to directly observe the resident selecting and performing the screening test. Specific curricula in patient-physician communication skills can easily incorporate a focus on screening for substance use conditions as one of the case examples. Screening and ongoing medical management of outpatients, referral to specialty treatment services and use of the electronic medical record-based screening and assessment systems are all learning opportunities that may be present at outpatient sites.

• *Emergency department*: Residents have the opportunity to assess patients with substance use conditions, and their consequences, both medical and social, and to identify existing local resources for referral to treatment. Residents will also see acute overdose and intoxication in this setting.

• *Medical consultation rotations/curriculum*: Residents may assist in the management of withdrawal syndromes on non-medical services, address peri-operative issues of patients with substance use conditions, as well as the medical complications of addictions.

• *Specialty addiction treatment experiences*: We recommend that all residents experience at least one of the following specialty addiction treatment settings (to which they will refer patients, and from which they will receive patients), to broaden their view of addiction and recovery and to understand the specialty care perspective. These programs include detoxification programs, needle exchange and methadone programs, 12-step meetings, residential rehabilitation programs and intensive outpatient treatment settings. These may be incorporated into an existing elective or ambulatory block, and need not be lengthy to be effective [56]. Residents' experience with substance use conditions would be further enriched by allowing residents to see patients in these settings, especially those in recovery who are doing well (who are often unlike patients residents recall from their internal medicine settings and experiences).

#### Evaluation of the Curriculum

The ability to demonstrate the achievement of competency-based learning objectives provides evidence that, when training is complete, graduating resident physicians can meet the health needs of the public [57]. In concordance with the ACGME's focus on *outcomes*, evaluation modalities should focus on whether and how fully internal medicine residents are incorporating concepts of addiction medicine into their practice, and whether residents are competently performing specific skills (e.g., screening and brief intervention) documented by direct observation and/or evidence in work products. Dimensions of evaluation of the unhealthy substance use curriculum include:

• Assessment of the effectiveness of didactic teaching

• Assessment of resident skill acquisition

• Clinical performance assessment and feedback

• Resident self-assessment and reflection

• Documentation of academic work products

##### Evaluation of the didactic sessions

To measure learning in didactic presentations, before-after ("pre-post") measures can be administered with each conference to detect changes in targeted knowledge, skills (or perceived skills), and attitudes. Future intentions can also be measured to determine if teaching influenced residents' plans for changes or improvements in clinical practice. However, sometimes pre- post testing is difficult due both to time pressures and the common tendency for some residents to arrive to conferences late and others to leave early. Audience response technology, such as TurningPoint^® ^(http://www.turningtechnologies.com accessed October 19, 2008), could be used as an alternative thereby integrating testing into an interactive presentation. This method would allow for the collection of real-time data using multiple choice questions near the beginning and the end of a teaching session. The quality and usefulness of the teaching can also be evaluated at the end of the didactic session employing a short resident questionnaire. Such evaluations are best kept to 3-5 questions to improve response rate.

##### Direct observation of resident skills

A "mini-CEX (Clinical Evaluation Exercise)"-style evaluation card can be designed specifically for observation and evaluation of key addiction medicine clinical skills. The mini-CEX is a snapshot of doctor/patient interaction, designed to assess the clinical skills, attitudes and behaviors of trainees essential to providing high quality care by supervisors observing an actual clinical encounter. Not all elements need be assessed on each occasion. Specific unhealthy substance use clinical skills can easily be incorporated into routinely performed CEXs. The mini-CEX approach to resident clinical skills assessment is a feasible, reliable, valid, and widely used evaluation method [58]. For unhealthy substance use patients, the mini-CEX would be customized to focus on key substance use condition interactions and patient cases and different evaluation cards could be customized for both the inpatient and ambulatory setting.

If the appropriate equipment is available in the clinic setting and patient consent is obtained, video recording of encounters where screening or other targeted addiction medicine skills are employed provides the resident with the opportunity to self-assess and provides the faculty preceptor the opportunity to offer detailed feedback on resident performance. The key is to set aside sufficient time with resident and preceptor to review the video interaction in detail, stop and start the interaction frequently for reflection and skill assessment, and construct a dialogue focusing on reinforcement of skillful performance and opportunities for performance improvement.

##### Performance measures

Teaching clinics may also opt to collect data on total number of patients screened and other individual resident performance statistics (e.g., brief intervention, referral to treatment). Data can be collected via medical record review and fed back to the resident. Key record indicators that can be measured include documentation of screening results, documentation of brief intervention provided, referrals, and follow-up.

##### Peer record reviews

Peer medical record review can be a successful teaching strategy. Working in dyads, each resident conducts an annual review of a record of a patient with unhealthy substance use (or a patient screened for the condition)[59]. Residents select the record for their colleague to review, and together, they prepare a presentation for a pre-clinic conference, identifying challenges, treatment options, and community resources. In the process, residents have opportunity for reflection, self-assessment, and specific feedback on their approach to care and/or management.

##### Objective Structured Clinical Exams (OSCEs)

The OSCE is an evaluation methodology where standardized patients (actors playing patient roles) are placed in mock healthcare settings to assess residents' clinical skills. Standardized patients are trained to portray specific patient case scenarios in a standardized manner [60] so that clinical challenges can be consistently presented to the participating residents. OSCEs using standardized patients are employed for clinical skills assessment in many US residency programs and most US medical schools [61-63]. For these exams, residents engage in a clinical interaction with the standardized patient that is observed and/or video/audio-recorded for skill assessment by faculty. Standardized patients can also be trained to provide reliable assessments of residents' performance. The ACGME has identified OSCEs as the most desirable method for assessing interviewing, communication and counseling skills, and preventive health procedures, [64] and therefore can be an effective way to assess specific clinical skills key to substance use condition-related patient care. OSCEs are most cost-effective when a large number of residents participate during the same administration [64]. Resources needed for effective OSCE administration are substantial and include: space (a clinical facility or OSCE center), cases designed to call for the use of targeted unhealthy substance use skills, checklists for faculty and/or patients to use to assess targeted skills, trained standardized patients, and sufficient skilled faculty for observation or review of recorded interactions.

##### Portfolio submissions

Portfolios are employed in resident evaluation for the documentation of clinical performance to meet competency criteria, documentation of program-related competence development, and documentation and tools for professional growth [65]. They are also commonly used to provide in-depth personalized mentoring to residents. There are a variety of different kinds of portfolios currently in use in residency training. Although many portfolios are paper, digital portfolios have also been shown to be well received in medical school and other settings, [66,67] as well as in residency training [68-71]. Digital portfolios can be employed not only to collect evidence of proficiency and professional growth, but can also enhance access and portability, organization, collaboration, and feedback [72].

For documentation and evaluation of addiction medicine skills, video recorded patient encounters on digital video discs (DVDs) or in digital format can be collected and maintained in the resident's academic portfolio. Pre-clinic conference presentations (slides, handouts, and presentation notes) can also be included. De-identified substance use condition management plans and reflective writings about related patient experiences or observations at AA meetings can also be valuable additions to resident portfolios. Portfolios are typically examined by program directors for the resident's annual review, are used as a basis for feedback on performance and resident professional development, and can be an important source of information for resident letters of recommendation for fellowship training or transition to private practice upon graduation.

#### Resources and implementation

##### Resources

The delivery of the core curriculum, through our proposed modules would consist of, at minimum, 12 hours of didactics, over three years of residency training. Residents' same clinical experiences during standard internal medicine rotations would provide the clinical reinforcement of skills, and solidify the key concepts of addiction medicine/unhealthy substance use. Additional focused evaluation activities will reinforce both didactic and clinical teaching and provide outcome measures required by the ACGME. This time commitment represents only a fraction of the focus received by cardiology and diabetes care.

##### Costs

Once a generation of internal medicine residency training program faculty with expertise sufficient to teach addiction medicine exist, teaching the required competencies to address unhealthy substance use should not require funds beyond those required for the whole program. Residency training programs often benefit from in-kind support or external support for special teaching efforts. Such added support would be useful for supporting experiences such as travel to treatment programs or standardized patients. However, these are the sorts of costs that, as for teaching of other competencies in other areas of medicine, are anticipated to be integrated with general funds for residency training, as part of the whole training program budget.

Achieving an adequate supply of internal medicine faculty capable of teaching unhealthy substance use competencies will be a cost. Unlike internal medicine subspecialty faculty, who are trained in medicine subspecialty fellowships and well-represented among internal medicine residency program faculty, medicine faculty with expertise in addiction medicine are not similarly well-represented. Such faculty will either need to come from new residency graduates, or from training current faculty, and in fairly large numbers. This training could occur by self-learning or specific continuing education experiences for existing faculty, or in national multidisciplinary training programs as have existed in the past and have been proposed recently (e.g., Centers of Excellence) [53].

##### Resources for Program Directors

For training existing faculty, residency programs could link with local addiction treatment programs, and the few internal medicine or other faculty with this expertise in academic medical centers. Continuing medical education programs in person (e.g., as listed at http://www.motivationalinterview.org/ for motivational interviewing (accessed February 14, 2010), and perhaps more efficiently, online, exist for training faculty. For example, the Alcohol Clinical Training Project (http://www.mdalcoholtraining.org accessed February 14, 2010) is a free resource for training faculty consisting of slides with case-based video vignettes, speaker notes and learner evaluation materials. The curriculum is flexible and modifiable and can be taught using all the components together in a 3-hour workshop or by using various components separately in 45 minute sessions (i.e., preclinic conference or attending rounds) or for self learning. The curriculum has been evaluated [37]. A related web publication ("Alcohol, other drugs and health: current evidence) can be used to for faculty (and residents) to keep up to date (http://www.aodhealth.org accessed February 14, 2008). Many teaching materials and information resources are available at http://www.nida.nih.gov, http://www.niaaa.nih.gov, and http://www.samhsa.gov (all accessed February 14, 2010). The NIAAA web site offers a clinician's guide to Helping Patients Who Drink Too Much and related continuing education materials (including slides and training videotapes).

##### Number of faculty needed

Residency programs will need to have sufficient faculty (depending on the number of residents in the program) to assure their residents receive training, both to deliver the didactics, but even more importantly to mentor residents and serve as role models. Faculty serving as preceptors may need to be trained in the knowledge and skills slated for this curriculum. Thus faculty development efforts must be implemented, particularly when employing an integrated model. Residents value skill and competence in their teachers, and multifaceted teachers - those who have an excellent grasp on medicine, will be more effective teachers about unhealthy substance use for their residents.

##### Expertise

Faculty teaching about unhealthy alcohol and drug use will need to have the competencies they are teaching (as noted previously). These faculty need not have addiction specialty expertise, and the expertise needed to address all competencies may be spread over a number of individuals. For example, one faculty member may have expertise in the management of inpatient alcohol withdrawal, another may be expert at screening and brief intervention. Many of the relevant competencies are similar to those that medicine faculty already must have to teach preventive medicine. These similar competencies are those that involve an understanding of modifiable risk factors and behaviors that risk chronic illnesses that are addressed by behavioral intervention aimed at lifestyle change (such as identification and addressing of depressive symptoms, medication nonadherence, physical inactivity). Such competencies are those reflected by ACGME competencies as communication skills (e.g., motivational counseling, for example is needed to address unhealthy drug use as well as lifestyle change and medication adherence). More teachers are needed to address competencies that all internists should have (e.g., prevention, screening, brief intervention, recognition of comorbidity, and ability to refer for pharmacotherapy and specialty care), whereas fewer teachers would be needed to address competencies needed to provide more specialized services such as prescription of buprenorphine for opioid dependence.

##### Required qualifications

Recently, the American Board of Addiction Medicine was established to examine and certify diplomats (http://www.abam.net/ accessed February 24, 2010). While not yet recognized by the American Board of Medical Subspecialties (ABMS) it is the only US medical specialty board that certifies addiction medicine physicians across a range of medical specialties. The ABMS does recognize the specialty of addiction psychiatry (certification available to neurologists and psychiatrists) and the American Osteopathic Boards of Anesthesiology, Family Medicine, Internal Medicine and Neurology and Psychiatry recognize added qualifications in addiction medicine for their diplomates. Training and certification but not board certification can be obtained in other ways by (doctor of medicine) internists. In addition, much of the knowledge relevant to internists--the management of patients with unhealthy alcohol and other drug use in general populations and health settings--is not traditionally the focus of addiction specialty training (of note many leaders in the field of education and research on screening and brief intervention in the past 40 years have been generalist physicians).

The first qualification for training internal medicine residents is that the teachers be internal medicine physicians. It is important for internists to teach this (in distinction to psychiatrists or non-physicians) for several reasons. First, resident physicians see their mentors and attending physicians modeling appropriate care of patients with alcohol and drug problems. Second, internists are the most appropriate teachers to tailor the broad knowledge and evidence related to these conditions to the content most relevant for medicine residents and to the teaching venues most appropriate. Lastly, medicine teachers are most likely to demonstrate role responsibility for these conditions (and, as such, residency programs will take such responsibility). Interdisciplinary care and teaching is critical in the area of substance use conditions and faculty from other disciplines should be involved. Residents will most likely be receptive to such teaching when introduced by their primary clinical teachers (as other specialty subjects are taught during their residencies). There are parallels for this model in other areas of internal medicine such as depression and diabetes care.

Specialty credentials are not required for medicine faculty teaching about unhealthy SU. However, continuing education courses and certification offered by the American Society of Addiction Medicine (ASAM) and attendance at professional continuing education meetings such as that of the Association for Medical Education and Research in Substance Abuse (AMERSA) can be helpful resources. Physicians teaching buprenorphine treatment of opioid dependence should be waivered by the Drug Enforcement Agency (DEA) by completing the required 8 hours of training or the other routes to achieve this goal (http://buprenorphine.samhsa.gov/index.html, accessed February 25, 2010). There have been numerous internists who have led teaching and educational efforts in internal medicine nationwide who have been successful without addiction specialty credentials or training. These teachers gain their expertise via generalist fellowships or focus on substance use during their residency training or via self- (by reading, online materials and workshops and courses at continuing education venues) and mentored teaching in their years as faculty.

##### Challenges in implementing new curricula in residency programs

Internal medicine educators are bombarded with teaching requirements, for example in genetics, ethics, communication skills, molecular medicine, geriatrics, sexual health, computer literacy, and interdisciplinary team based care, to mention a few. These demands reflect continued rapid growth in scientific knowledge coupled with society's expectation that physicians minister to social and psychological as well as physical infirmities[73]. Training programs are challenged to produce competent, ethical and caring physicians in a defined time period of three years, but must address key health care needs of the population, among which unhealthy substance use certainly figures highly. As internal medicine educators, we cannot afford to ignore addiction medicine with the significant national burden of disease as well as proven treatment interventions. There is still a serious mismatch between what we know to be good quality care and the care that is actually delivered, [52] and residency programs are in a position to fill this gap. One way to address the challenge of a bursting curricula in residency training, is to integrate a new focus of discussion into standard teaching venues.

##### Champions

For any curriculum change to be implemented and maintained there needs to be a motivated and vigilant faculty champion who is dedicated to providing appropriate intervention and support to the residency program as needed. Ideally, this champion would be a faculty member who is active in curriculum management and reform and willing to take on a leadership role. Designing an unhealthy substance use curriculum implementation strategy for any residency program will require a fundamental appreciation of the culture and resources of the program, its faculty, and its teaching venues, as well as an understanding of the addiction field itself. The champion would probably be active in teaching several didactic sessions to both residents and faculty as needed, and would monitor the status of the curriculum and its evaluation to fine-tune and improve the program over time.

## Summary

Given the burden of disease and the effective interventions that can be delivered to patients by internal medicine physicians, teaching about unhealthy substance use must, and can be incorporated into internal medicine residency training. In part because clinical management for substance use conditions is most effective when integrated with medical and other care, education should mirror this approach, integrating curricula on unhealthy substance use into overall residency training. This integration is not particularly resource intensive and can be done by making use of many existing teaching venues. Perhaps the biggest challenge will be initially getting sufficient trained internal medicine faculty with expertise to teach the curriculum for residents. Getting sufficient faculty will likely require resources since unlike other medicine topics, a new faculty (or new expertise, or at least expertise that is not in abundant supply in internal medicine residency programs) is required. Many educational resources already exist to achieve this goal though the manpower and time for training will still require investment. Federally supported faculty development efforts have trained a small number of expert teachers. Programs similar to these will likely be needed to implement curricula such as we propose. Further, the development of addiction medicine as a specialty by the American Board of Addiction Medicine is likely to support implementation of such curricula in the future. With adequate faculty, few other challenges remain. Core unhealthy substance use competencies for physicians, easily adapted to internists, have been established, and they can contribute to meeting ACGME general competencies. A curriculum has been outlined herein, based on this guidance. Evaluation of outcomes is fairly straightforward and achievable. Accrediting bodies can have a significant role in improving the teaching and therefore care for patients with unhealthy substance use. The time is right to improve (and require excellence in) residency training about unhealthy substance use in internal medicine residency training programs.

## Competing interests

The authors declare that they have no competing interests.

## Authors' contributions

AHJ, DPA and RS contributed equally to this work, CED prepared the Evaluation of the Curriculum section. All authors were involved in revising the manuscript, and all authors read and approved the final manuscript.

## Pre-publication history

The pre-publication history for this paper can be accessed here:

http://www.biomedcentral.com/1472-6920/10/22/prepub

## References

[B1] Saitz R Unhealthy alcohol use N Engl J Med 2005 352 596 607 10.1056/NEJMcp042262 15703424

[B2] Cherry DK Woodwell DA Rechtsteiner EA National Ambulatory Medical Care Survey: 2005 Summary. Advance data from vital and health statistics Hyattsville, MD 2007 387 17703793

[B3] SAMHSA Results from the 2006 National Survey on Drug Use and Health: National Findings Office of Applied Studies NSDUH Series H-32, DHHS Publication No. SMA 07-4293. Rockville, MD 2007

[B4] Office of National Drug Control Policy National Drug Control Strategy. Data Supplement Washington, D.C 2005 http://www.whitehousedrugpolicy.gov/publications/policy/ndcs09/ndcs09_data_supl/ds_drg_rltd_tbls.pdf Accessed October 19, 2008

[B5] Mokdad AH Marks JS Stroup DF Gerberding JL Actual causes of death in the United States, 2000 JAMA 2004 291 1238 1245 10.1001/jama.291.10.1238 15010446

[B6] NIAAA 2008 http://www.niaaa.nih.gov/Resources/DatabaseResources/QuickFacts/EconomicData/cost8.htm Access year August 25, 2008

[B7] Greenlund KL Giles WH Keenan NL Ward J, Warren C Heart disease and stroke mortality in the 20th century Silent Victories: The History and Practice of Public Health in Twentieth Century America 2006 Oxford, England.: Oxford University Press

[B8] Rosamond W Flegal K Friday G Heart disease and stroke statistics -- 2007 update: a report from the American Heart Association Statistics Committee and Stroke Statistics Subcommittee Circulation 2006 113 169 171 10.1161/CIRCULATIONAHA.106.179918 17194875

[B9] Fiellin D Butler R D'Onofrio G Brown R O'Connor P The physician's role in caring for patients with substance use disorders: Implications for medical education and training Subst Abuse 2002 23 207 212 10.1080/08897070209511516 23580996

[B10] Michaud CM Murray CJ Bloom BR Burden of disease -- implications for future research JAMA 2001 285 535 539 10.1001/jama.285.5.535 11176854

[B11] Office of National Drug Control Policy The economic cost of drug abuse in the United States, 1992-2002 Washington, D.C 2002 http://www.ncjrs.gov/ondcppubs/publications/pdf/economic_costs.pdf Accessed August 25, 2008

[B12] SAMHSA Results from the 2006 national survey of drug use and health: national findings http://www.drugabusestatistics.samhsa.gov/NSDUH/2k6NSDUH/2k6results.cfm Accessed August 25, 2008

[B13] Friedmann PD Saitz R Samet JH Management of adults recovering from alcohol or other drug problems: relapse prevention in primary care JAMA 1998 279 1227 1231 10.1001/jama.279.15.1227 9555766

[B14] Lewis DC Despite benefit, physicians slow to offer brief advice on harmful alcohol use JAMA 1996 299 751 753 10.1001/jama.299.7.751 18285582

[B15] McLellan AT Lewis DC O'Brien CP Kleber HD Drug dependence, a chronic medical illness: implications for treatment, insurance, and outcomes evaluation JAMA 2000 284 1689 1695 10.1001/jama.284.13.1689 11015800

[B16] Saitz R Horton NJ Larson MJ Winter M Samet JH Primary medical care and reductions in addiction severity: a prospective cohort study Addiction 2005 100 70 78 10.1111/j.1360-0443.2005.00916.x 15598194

[B17] Samet JH Friedmann PD Saitz R Benefits of linking primary medical care and substance abuse services: patient, provider, and societal perspectives Arch Intern Med 2001 161 85 91 10.1001/archinte.161.1.85 11146702

[B18] Solberg LI Maciosek MV Edwards NM Primary care intervention to reduce alcohol misuse: ranking its health impact and cost effectiveness Am J Prev Med 2008 34 2 143 152 10.1016/j.amepre.2007.09.035 18201645

[B19] Maciosek MV Coffield AB Edwards NM Flottemesch TJ Goodman MJ Solberg LI Priorities among effective clinical preventive services: results of a systematic review and analysis Am J Prev Med 2006 31 1 52 61 10.1016/j.amepre.2006.03.012 16777543

[B20] Maciosek MV Coffield AB Edwards NM Goodman MJ Flottenmesch TJ Solberg LL Priorities among effective clinical preventive services: results of a systematic review and analysis Am J Prev Med 2006 31 1 52 61 10.1016/j.amepre.2006.03.012 16777543

[B21] McCormick KA Cochran NE Back AL Merrill JO Williams EC Bradley KA How primary care providers talk to patients about alcohol: a qualitative study J Gen Intern Med 2006 21 966 972 10.1007/BF02743146 16918743 PMC1831591

[B22] Kuehn BM Despite benefit, physicians slow to offer brief advice on harmful alcohol use JAMA 2008 299 751 753 10.1001/jama.299.7.751 18285582

[B23] Vastag B Addiction poorly understood by clinicians: experts say attitudes, lack of knowledge hinder treatment JAMA 2003 290 1299 1303 10.1001/jama.290.10.1299 12966106

[B24] Saitz R Friedmann PD Sullivan LM Professional satisfaction experienced when caring for substance-abusing patients: faculty and resident physician perspectives J Gen Intern Med 2002 17 373 376 12047735 10.1046/j.1525-1497.2002.10520.x PMC1495049

[B25] Stimmel B Cohen D Swartz M An assessment of house staff's knowledge of alcohol and substance abuse utilizing standardized patients Subst Abuse 2000 21 1 7 10.1080/08897070009511413 12466643

[B26] Institute of Medicine Improving the quality of health care for mental and substance-use conditions 2006 Washington, DC, The National Academics Press 20669433

[B27] Conigliaro J Delos Reyes C Parran TV Schulz JE Graham AW, Schultz TK, Mayo-Smith MF, Ries RK, Wilford BB Principles of screening and early intervention Principles of Addiction Medicine 2003 Third Chevy Chase: American Society of Addiction Medicine

[B28] Office of National Drug Control Policy Leadership Conference on Medical Education in Substance Abuse December 1-2, 2004 (report) Washington, D.C 2004 http://www.ncjrs.gov/ondcppubs/publications/pdf/medical_educ_2004.pdf Access date, October 19

[B29] Fleming MF Barry KL Davis A Kropp S Kahn R Rivo M Medical education about substance abuse: changes in curriculum and faculty between 1976 and 1992 Acad Med 1994 69 362 369 10.1097/00001888-199405000-00009 8166918

[B30] Fleming MF Manwell LB Kraus M Isaacson JH Kahn R Stauffacher EA Who teaches residents about the prevention and treatment of substance use disorders? A national survey J Fam Pract 1999 48 725 729 10498080

[B31] Foley ME Garland E Stimmel B Merino R Innovative clinical addiction research training track in preventive medicine Subst Abuse 2000 21 111 119 10.1080/08897070009511423 12466651

[B32] Kuehn BM Centers to weave addiction treatment into medical education JAMA 2007 297 1763 10.1001/jama.297.16.1763 17456812

[B33] Miller NS Sheppard LM Colenda CC Magen J Why physicians are unprepared to treat patients who have alcohol- and drug-related disorders Acad Med 2001 76 410 418 11346513 10.1097/00001888-200105000-00007

[B34] Wyatt SA Vilensky W Manlandro JJ Jr Dekker MA Medical education in substance abuse: from student to practicing osteopathic physician J Am Osteopath Assoc 2005 105 S18 S25 16118358

[B35] Klamen DL Miller NS Integration in education for addiction medicine J Psychoactive Drugs 1997 29 263 268 9339858 10.1080/02791072.1997.10400200

[B36] Project Mainstream Improving substance abuse education for health professionals http://www.projectmainstream.net Accessed March 11, 2008

[B37] Alford DP Richardson JM Chapman SE Dube CE Schadt RW Saitz R A web-based alcohol clinical training (ACT) curriculum: Is in-person faculty development necessary to affect teaching? BMC Medical Education 2008 8 11 10.1186/1472-6920-8-11 18325102 PMC2329623

[B38] Parish SJ Ramaswamy M Stein MR Sachur EK Arnsten JH Teaching about substance abuse with objective structured clinical exams J Gen Intern Med 2006 21 453 459 10.1111/j.1525-1497.2006.00426.x 16704387 PMC1484780

[B39] Alford DP Compton P Samet JH Acute pain management for patients receiving maintenance methadone or buprenorphine therapy Ann Intern Med 2006 144 127 134 16418412 10.7326/0003-4819-144-2-200601170-00010 PMC1892816

[B40] Samet JH Rollnick S Barnes H Beyond CAGE. A brief clinical approach after detection of substance abuse Arch Intern Med 1996 156 2287 2293 10.1001/archinte.156.20.2287 8911235

[B41] Abrams Weintraub T Saitz R Samet JH Education of preventive medicine residents: alcohol, tobacco, and other drug abuse Am J Prev Med 2003 24 1 101 105 10.1016/S0749-3797(02)00567-6 12554029

[B42] Isaacson JH Fleming MF Kraus M Kahn R Mundt M A national survey of training in substance use disorders in residency programs J Stud Alcohol 2000 61 912 915 11188498 10.15288/jsa.2000.61.912

[B43] Chossis I Lane C Gache P Michaud PA Pecoud A Rollnick S Daeppen JB Effect of training on primary care residents' performance in brief alcohol intervention: a randomized controlled trial J Gen Intern Med 2007 22 8 1144 1149 10.1007/s11606-007-0240-2 17541671 PMC2305743

[B44] Karam-Hage M Nerenberg L Brower KJ Modifying residents' professional attitudes about substance abuse treatment and training? Am J Addict 2001 10 40 47 10.1080/105504901750160466 11268827

[B45] Geller G Levine DM Mamon JA Moore RD Bone LR Stokes EJ Knowledge, attitudes, and reported practices of medical students and house staff regarding the diagnosis and treatment of alcoholism JAMA 1998 261 3115 3120 10.1001/jama.261.21.3115 2716143

[B46] Madden TE Graham AV Straussner SL Interdisciplinary benefits in Project MAINSTREAM: a promising health professions educational model to address global substance abuse J Interprof Care 2006 20 655 664 10.1080/13561820600893890 17095443

[B47] Marcus MT Brown RL Straussner SL Creating change agents: a national substance abuse education project Subst Abuse 2005 26 5 15 10.1300/j465v26n03_03 16837406

[B48] Madras BK Compton WM Avula D Stegbauer T Stein JB Clark HW Screening, brief interventions, referral to treatment (SBIRT) for illicit drug and alcohol use at multiple healthcare sites: comparison at intake and 6 months later Drug Alcohol Depend 2009 99 1-3 280 295 10.1016/j.drugalcdep.2008.08.003 18929451 PMC2760304

[B49] Physician Quality Reporting Initiative Centers for Medicare and Medicaid Services http://www.cms.hhs.gov/pqri Accessed October 28, 2009

[B50] Candidate Measure Profile for Assessing and Treating Tobacco, Alcohol, and Other Drug Use and Dependence http://www.jointcommission.org/NR/rdonlyres/3528C77F-7BB5-4D62-85E9-87C8285D8652/0/CandidateFinalMIFTADD6.doc accessed September 29, 2009

[B51] Yoast RA Wilford BB Hayashi SW Encouraging physicians to screen for and intervene in substance use disorders: Obstacles and strategies for change J Addict Dis 2008 27 3 1299 1303 10.1080/10550880802122687 18956531

[B52] Greiner AC Knebel E (Eds) Committee on the Health Professions Education Summit 2003 Washington, D.C.: National Academics Press 25057657

[B53] Haack ML Adger HA (Eds) Strategic Plan for Interdisciplinary Faculty Development; Arming the Nation's Health Professional Workforce for a New Approach to Substance Use Disorders 2002 23

[B54] Whitlock EP Polen MR Green CA Orleans T Klein J Behavioral counseling interventions in primary care to reduce risky/harmful alcohol use by adults: a summary of the evidence for the U.S. Preventive Services Task Force Ann Intern Med 2004 140 7 557 568 15068985 10.7326/0003-4819-140-7-200404060-00017

[B55] Skinner HA The drug abuse screening test Addict Behav 1982 7 4 363 367 10.1016/0306-4603(82)90005-3 7183189

[B56] Rose AJ Stein MR Arnsten JH Saitz R Teaching internal medicine resident physicians about alcoholics anonymous: A pilot study of an educational intervention Subst Abuse 2006 26 3 5 11 10.1300/J465v26n03_03 PMC1803081 17135175

[B57] Lynch DC Swing SR Research Department ACGME Key Considerations for selecting assessment instruments and implementing assessment systems 2008 http://www.acgme.org/outcome/assess/keyConsider.asp accessed October 29, 2008

[B58] Norcini JJ Blank LL Duffy DF Fortna GS The Mini-CEX: a method for assessing clinical skills Ann Intern Med 2003 138 476 481 12639081 10.7326/0003-4819-138-6-200303180-00012

[B59] Gillig PM Barr A A model for multidisciplinary peer review and supervision of behavioral health clinicians Community Mental Health J 1999 35 4 361 365 10.1023/A:1018766008133 10452702

[B60] Stimmel B (Ed) Utilizing standardized patient protocols to improve skills in identifying tobacco, alcohol and other drug use: A manual of cases 1998 New York: Josiah Macy Jr. Foundation

[B61] ACGME/ABMS Joint Initiative Suggested best methods for evaluation. Attachment/Toolbox of Assessment Methods. version 1.1 2000 http://www.acgme.org/outcome/assess/toolbox.asp Accessed 10/16/08

[B62] Stillman PL Swanson DB Regan MB Assessment of clinical skills of residents utilizing standardized patients. A follow-up study and recommendations for application Ann Intern Med 1991 114 5 393 401 1992883 10.7326/0003-4819-114-5-393

[B63] Stillman PL Swanson DB Smee S Assessing clinical skills of residents with standardized patients Ann Intern Med 1986 105 5 762 771 3767153 10.7326/0003-4819-105-5-762

[B64] ACGME Outcomes Project, American Board of Medical Specialties Toolbox of assessment methods: A product of the joint initiative 2000 http://www.acgme.org/Outcome/assess/ToolTable.pdf Accessed 10/16/08

[B65] Tillema H Portfolios as developmental assessment tools Int J Training Dev 2001 5 2 126 135 10.1111/1468-2419.00127

[B66] Dreissen EW Muijtjens AM van Tartwijk J Vleuten CP van der Web- or paper-based portfolios: is there a difference? Med Educ 2007 41 1067 1073 10.1111/j.1365-2923.2007.02859.x 17973767

[B67] Woodward H Nanlohy P Digital portfolios: fact or fashion? Assess Eval Higher Educ 2004 29 2 227 238 10.1080/0260293042000188492

[B68] Carraccio C Englander R Evaluating competence using a portfolio: a literature review and web-based application to the ACGME competencies Teaching Learning Med 2004 16 4 381 387 10.1207/s15328015tlm1604_13 15582877

[B69] Clay AS Petrusa E Harker M Andolsek K Development of a web-based, specialty specific portfolio Med Teacher 2007 29 311 316 10.1080/01421590701291428 17786743

[B70] Fung MFKF Walker M Fung KFK An internet-based learning portfolio in resident education: The KOALATM multi-centre programme Med Educ 2000 34 474 479 10.1046/j.1365-2923.2000.00571.x 10792690

[B71] Rosenberg ME Watson K Paul J Miller W Harris I Valdivia TD Development and implementation of a web-based evaluation system for an internal medicine residency program Acad Med 2001 6 92 95 10.1097/00001888-200101000-00024 11154204

[B72] May K Lowry B Digital Portfolios to Support Professional Development and Program Evaluation 2007

[B73] Batalden P Leach D Swing S Dreyfus H Dreyfus S General competencies and accreditation in graduate medical education Health Affairs 2002 21 5 103 111 10.1377/hlthaff.21.5.103 12224871

